# The Efficacy of Short-term Gated Audiovisual Speech Training for Improving Auditory Sentence Identification in Noise in Elderly Hearing Aid Users

**DOI:** 10.3389/fpsyg.2017.00368

**Published:** 2017-03-13

**Authors:** Shahram Moradi, Anna Wahlin, Mathias Hällgren, Jerker Rönnberg, Björn Lidestam

**Affiliations:** ^1^Linnaeus Centre HEAD, Department of Behavioral Sciences and Learning, Linköping University,Linköping, Sweden; ^2^Department of Otorhinolaryngology and Department of Clinical and Experimental Medicine, Linköping University,Linköping, Sweden; ^3^Department of Behavioral Sciences and Learning, Linköping University,Linköping, Sweden

**Keywords:** audiovisual training, auditory training, hearing aid, speech-in-noise identification

## Abstract

This study aimed to examine the efficacy and maintenance of short-term (one-session) gated audiovisual speech training for improving auditory sentence identification in noise in experienced elderly hearing-aid users. Twenty-five hearing aid users (16 men and 9 women), with an average age of 70.8 years, were randomly divided into an experimental (audiovisual training, *n* = 14) and a control (auditory training, *n* = 11) group. Participants underwent gated speech identification tasks comprising Swedish consonants and words presented at 65 dB sound pressure level with a 0 dB signal-to-noise ratio (steady-state broadband noise), in audiovisual or auditory-only training conditions. The Hearing-in-Noise Test was employed to measure participants’ auditory sentence identification in noise before the training (pre-test), promptly after training (post-test), and 1 month after training (one-month follow-up). The results showed that audiovisual training improved auditory sentence identification in noise promptly after the training (post-test vs. pre-test scores); furthermore, this improvement was maintained 1 month after the training (one-month follow-up vs. pre-test scores). Such improvement was not observed in the control group, neither promptly after the training nor at the one-month follow-up. However, no significant between-groups difference nor an interaction between groups and session was observed. Conclusion: Audiovisual training may be considered in aural rehabilitation of hearing aid users to improve listening capabilities in noisy conditions. However, the lack of a significant between-groups effect (audiovisual vs. auditory) or an interaction between group and session calls for further research.

## Introduction

Age-related hearing loss (Presbycusis) is one of the most common disorders in elderly people, and hearing loss prevalence is growing because of an aging population (Lin et al., 2011; World Health Organization, 2012). The most evident negative consequence of hearing loss is difficulty in perceiving speech, especially in adverse listening conditions (e.g., Needleman and Crandell, 1995). This can lead to other difficulties such as social isolation, particularly in women (Mick et al., 2014); mental health problems (e.g., depression, anxiety; Li et al., 2014; Keidser et al., 2015); spouse relationship difficulties (Scarinci et al., 2012); and reduction in quality of life (Dalton et al., 2003).

Currently, the most common method to compensate for the speech perception difficulties of sensorineural hearing-impaired listeners is to prescribe hearing aids. Despite the use of advanced digital hearing aids, our previous findings have shown that elderly hearing aid users have inferior performance compared to their age-matched counterparts with normal hearing in perceiving speech stimuli when a prior semantic context is lacking (Moradi et al., 2014, 2016). In addition, independent studies have shown that the amplification of sounds alone cannot fully restore difficulties in the auditory perception of speech stimuli in people with hearing loss (Dimitrijevic et al., 2004; Ahlstrom et al., 2014; Moradi et al., unpublished). As a consequence, people with hearing loss need other methods of rehabilitation, in addition to hearing aids, to compensate more fully for their difficulties in perceiving speech stimuli.

Auditory training has been reported to be an effective method in people with hearing loss to improve phoneme recognition (e.g., Stecker et al., 2006; Ferguson et al., 2014) and word recognition (e.g., Burk et al., 2006). In sentence recognition, however, the current research with regards to the effect of auditory training on subsequent sentence identification in noise is inconclusive. For instance, Levitt et al. (2011) and Rao et al. (2017), both using ReadMyQuips auditory training program, and Sweetow and Sabes (2006) and Olson et al. (2013), using Listening and Communication Enhancement auditory training program, demonstrated the efficiency of auditory training on auditory sentence identification in noise, in hearing-aid users. However, Bock and Abrams (2013) and Abrams et al. (2015) reported no effects of auditory training on sentence identification in noise in hearing aid users.

Moradi et al. (2013), using a gating paradigm, studied the extent to which the addition of visual cues to an auditory speech signal facilitates the identification of speech stimuli in both silence and noise in young normal-hearing listeners. In the gating paradigm, participants are presented with successive fragments of a given speech stimulus (e.g., a word) and their task is to suggest a word that can be a continuation to that presented fragments (Grosjean, 1980). The main purpose of the gating paradigm is to estimate the isolation point, which is the shortest time from the onset of a speech token that is required for correct identification. A secondary finding by Moradi et al. (2013) was that the participants who were first exposed to gated audiovisual speech stimuli (consonants, words, and words embedded within sentences, presented in both silence and noise) subsequently had better performance in a Swedish version of HINT (Hällgren et al., 2006) than those who were first exposed to those speech stimuli, but in an auditory-only modality. In order to investigate whether the secondary finding by Moradi et al. (2013) was a genuine effect, Lidestam et al. (2014) conducted a randomized control group study. Young participants with normal hearing were randomly divided into three training groups: gated audiovisual speech training group, gated auditory speech training group, and a control group that simply watched a movie clip. The auditory and audiovisual speech training tasks consisted of identifying gated consonants and words presented audiovisually or aurally. Their results replicated the finding by Moradi et al. (2013) by showing that only the participants in the audiovisual training group experienced an improvement in HINT performance, while the participants in the auditory-only training and control groups did not. Interestingly, the findings of these two studies showed that the effect of audiovisual training on improving sentence comprehension in noise was independent of the idiosyncrasies of the talker’s articulation, as the talkers in the training and the HINT were not the same. This finding differs from cross-modal studies which have shown that familiarity with the talkers is a key factor in subsequent improvement in auditory speech processing (Rosenblum et al., 2007; von Kriegstein et al., 2008; Wu et al., 2013). Together, it seems that providing audiovisual training materials in a gating format is an efficient short-term approach in improving auditory speech-in-noise identification. This is most likely due to gated audiovisual speech stimulation tunes the attentional resources with richer speech input that subsequently reinforces the auditory map to phonological and lexical representations in the mental lexicon.

The present study is an extension of the study by Lidestam et al. (2014), which focuses on experienced hearing aid users. To do this, we measured the HINT scores of elderly hearing-aid users before the training (pre-test scores), immediately after the training (post-test scores), and 1 month after the training (one-month follow-up scores).

In the present study, we predict that the gated audiovisual speech training results in better performance of HINT in terms of SNR in elderly hearing aid users immediately after the training (post-test vs. pre-test comparison) and that this audiovisual training effect might be maintained in the follow-up measurement (one-month follow-up vs. pre-test comparison). We also predict no improvement from one session of gated auditory training on HINT in elderly hearing aid users either immediately after the training (post-test vs. pre-test comparison) or 1 month after the training (one-month follow-up vs. pre-test comparison). These predictions are based on our prior studies in normal hearing listeners which showed that one session of gated auditory training did not improve subsequent HINT performance, but gated audiovisual training did.

## Materials and Methods

### Participants

Twenty-five native Swedish speakers (16 men and 9 women) with a symmetrical bilateral mild to moderate hearing loss consented to participate in this study. They were recruited from an audiology clinic patient list at Linköping University Hospital, Sweden. Their ages at the time of testing ranged from 63 to 75 years (*M* = 70.8, *SD* = 2.9 years). They had been hearing aids users for at least 6 months. The participants were randomized into experimental (audiovisual training, *n* = 14, mean age = 71.8 years, 10 men and 4 women) and control (auditory training, *n* = 11, mean age = 69.5 years, 6 men and 5 women) groups by coin flipping (hence the unequal group sizes).

The participants wore various in-the-ear (ITE), behind-the-ear (BTE), completely-in-the-canal (CIC), and receiver-in-the-ear (RITE) digital hearing aids. The hearing aids were fitted for them in their most recent visit at Linköping University Hospital, based on each listener’s individual needs, by licensed audiologists who were independent of the present study. All of these hearing aids used non-linear processing and had been fitted according to manufacturer instructions. Participants wore their own hearing aids both in the training condition and in the HINT condition, with no change in the amplification setting of their own hearing aids throughout the study. **Table 1** shows the information about age ranges, type and brand of hearing aid, time since first hearing aid fitting, and the pure-tune average (PTA) thresholds across seven frequencies (125, 250, 500, 1000, 2000, 4000, and 8000 Hz) for the left and right ears for each participant in the experimental and control groups.

**Table 1:** Age ranges, time since first hearing fitting, PTA7 for each ear, and the type and brand of hearing aid of each participant in experimental and control groups.

Group	Participant	Age ranges (years)	Time since first hearing aid fitting (years)	PTA7 right ear (dB HL)	PTA7 left ear (dB HL)	Type and brand of hearing aid
Experimental group (Audiovisual speech training)	E1	70–75	9.6	40	35.7	BTE, Phonak, Ambra M H20
	E2	65–70	3.9	45	50	CIC, Widex, Mind 440 M4
	E3	70–75	9.8	33.6	37.9	BTE, Oticon, K140 13
	E4	70–75	1.8	30	31.4	BTE, Phonak Exelia Art Micro
	E5	70–75	0.8	39.3	33.6	BTE, Phonak, Audeo S Smart IX
	E6	70–75	1.2	37.9	45	BTE, Phonak, Ambra M H20
	E7	70–75	1.7	59.3	51.4	ITE, Oticon, K220
	E8	70–75	5.8	45.7	48.6	RITE, Oticon, Vigo Pro
	E9	65–70	3.8	45.7	45	ITE, Beltone, True9 35
	E10	70–75	4.3	45	45	BTE, Widex, Dream D4-XP
	E11	70–75	7.8	53.6	57.1	BTE, Oticon, Vigo Pro Power
	E12	65–70	0.9	45.7	46.4	BTE, Widex, Clear C4-9
	E13	70–75	7.7	50	51.4	ITE, Oticon, K220
	E14	65–70	3.5	43.4	49.2	BTE, Phonak Exelia Art Micro
Control Group (Auditory speech training)	C1	70–75	7.1	50.7	42.9	BTE, Oticon, K220 13
	C2	70–75	1.4	28.6	35	BTE, Oticon, EPOQ X W 13
	C3	70–75	2.5	32.1	33.6	BTE, Phonak, Versata Art Mico
	C4	70–75	1.2	39.3	37.9	BTE, Phonak, Ambra M H20
	C5	65–70	3.3	32.1	34.3	BTE, Phonak Exelia Art Micro
	C6	70–75	10.8	42.1	40.7	BTE, Phonak, Ambra Micro
	C7	70–75	1.1	42.1	40	BTE, Resound, Alera9 AL962-DVIRW
	C8	60–65	0.6	37.9	41.4	RITE, Oticon, K220 mini
	C9	65–70	18.4	57.1	58.6	BTE, Oticon, K220 13
	C10	65–70	1.9	37.9	44.3	BTE, Phonak Exelia Art Micro
	C11	70–75	1.0	55	53.6	BTE, Phonak Exelia Art Micro

The participants reported themselves to be in good health and had normal or corrected-to-normal vision with glasses.

The study inclusion criteria were as follows: (1) age over 50 years but less than 80 years; (2) Swedish as the native language; and (3) bilateral hearing loss with an average threshold of more than 35 dB HL for pure tone frequencies of 500, 1000, 2000, and 4000 Hz.

All participants were fully informed about the study and gave written consent for their participation in this study. In addition, they were paid (500 SEK) in return for their participation in this study. The Linköping regional ethical review board approved the study, including the informational materials and consent procedure.

### Speech Identification Training Conditions

**FIGURE 1:**
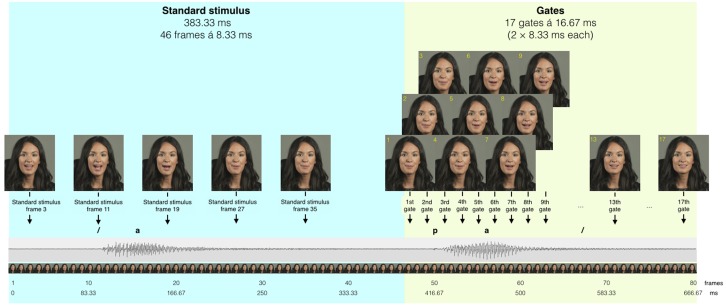
**An illustration of gated presentation of speech stimuli.** Reprinted from Moradi et al. (2016). Copyright 2016 by Sage Publications Inc. Adapted with permission.

The speech identification tasks comprised the same speech stimuli that were used by Lidestam et al. (2014). Participants were presented with gated Swedish consonants and words for both the audiovisual (experimental) and auditory-only (control) training conditions. **Figure 1** represents an example of the gated audiovisual consonant identification task.

In the audiovisual speech presentation, the talker’s hair, face, and shoulders were shown. The average overall sound pressure level (SPL) for the presentation of gated speech stimuli was set at 65 dB SPL, at a SNR of 0 dB, in both the auditory and audiovisual training conditions. This was measured in the vicinity of the participant’s head with a Larson Davis System 824 sound level meter (Provo, UT, USA) in a free field. The background noise was a steady-state broadband noise, from Hällgren et al. (2006), which was resampled and spectrally matched to the speech stimuli used in the present study. The onset and offset of background noise were simultaneous to the onset and offset of speech stimuli. The participants in the auditory training group received the same exact speech stimuli as the participants in audiovisual training group, but without visual input. Matlab (2010b) was used to associate the background noise to the auditory speech stimuli in a gating format at 0 dB SNR. To present speech stimuli in an audiovisual modality, Matlab and Psychophysics Toolbox (Brainard, 1997; Pelli, 1997; Kleiner et al., 2007) were used to synchronize auditory and visual speech stimuli. Detailed information about the synchronization of video and audio speech signal and the Matlab scripts used to gate them is available in Lidestam (2014).

### Consonants

Eighteen Swedish consonants were presented to participants in a vowel-consonant-vowel syllable format (/aba, ada, afa, aga, aja, aha, aka, ala, ama, ana, aŋa, apa, ara, aʈa, asa, a*∫*a, ata, and ava/) in the audiovisual and auditory-only training conditions. The gate size for consonants was set at 16.67 ms. Gating started after the first vowel (/a/) onset and at the beginning of the consonant onset. Hence, the first gate included the vowel /a/ plus the initial 16.67 ms of the consonant, the second gate provided an additional 16.67 ms of the consonant (a total of 33.33 ms), and so on. The consonant gating task took 10–15 min to complete.

### Words

Twenty-three Swedish monosyllabic words were presented to participants in a consonant-vowel-consonant (CVC) format (all nouns) in the audiovisual and auditory-only training conditions. The selected words had average to high frequencies according to the Swedish language corpus PAROLE [Språkbanken (The Swedish Language Bank), 2011] and also had a small-to-average number of phonological neighbors (three to six different words with similar articulation of the first two phonemes). For instance the word /dos/ has the neighbors /dog, dok, dop, don/. The gate size was 33.3 ms, similar to our previous studies (e.g., Moradi et al., 2013; Lidestam et al., 2014). The rationale for this gate size was based on our pilot findings, which showed that the identification of words with a gate size of 16.67 ms (starting from the first phoneme) in CVC format lead to exhaustion and loss of motivation. Hence, a double gate size (33.3 ms) starting from the onset of second phoneme was used to avoid fatigue in participants. The word gating task took 20–25 min to complete.

### Hearing-In-Noise Test

We used a Swedish version of the HINT (Hällgren et al., 2006) to measure participants’ sentence identification in noise ability. The HINT consisted of everyday sentences, from minimum three to maximum seven words in length on a background of steady-state speech-shaped noise. The first sentence in each list (in both the practice and experimental lists) was presented at 65 dB SPL and 0 dB SNR. The participants were asked to listen and repeat each sentence correctly. An automatic, adaptive up-down procedure was used to determine the SNR of each participant at a correct response rate of 50%. If all words were correctly repeated, the SNR was decreased by 2dB and if one or more words were not correctly repeated, the SNR was raised by 2 dB.

In the present study, the HINT scores of participants were collected in three sessions (pre-test, post-test, and one-month follow-up). Each session consisted of a practice list (with 10 sentences) and an experimental list (with 20 sentences). In each session, participants were first familiarized with the test using a 10-sentence practice list. To determine the SNR for each participant, a 20-sentence list was used in each session. Hence, 30 sentences in each session were used. There are 5 practice lists and 25 experimental lists in HINT. We chose three practice and three experimental lists that had the same SNRs based on norm data in normal hearing listeners (see Hällgren et al., 2006). To avoid repetition effects, we randomized the presentation of lists (practice and experimental lists) across participants in each training group, such that no list (or item) was repeated for any participant. The HINT took approximately 10 min to complete in each session.

It should be noted that the talkers of the HINT and training speech materials were different.

### Procedure

Participants were tested individually in a quiet room at Linköping University. They were sat in front of a Mitsubishi Diamond Pro 2070 SB cathode ray tube (CRT) monitor (Mitsubishi Electric, Tokyo, Japan). The monitor was turned off during auditory-only presentation. Auditory speech stimuli were delivered via an iMac computer, which was routed to the input of two loudspeakers (Genelec 8030A) located to the right and left of the CRT monitor. The experimenter used the iMac to present the gated stimuli and monitor the participants’ progress. There was a screen between the iMac monitor and the CRT monitor used for stimulus presentation, preventing participants from seeing the experimenter’s monitor and the response sheets.

The study was conducted over two separate sessions. The first session for both groups started with the pre-test measurement of participants’ HINT scores. Participants subsequently underwent gated audiovisual or auditory speech identification training. This entailed a practice session to allow participants to become familiarized with the gated presentation of stimuli. The practice session comprised three gated consonants (/v k ŋ/) and two gated words (/tum [inch]/ and /bil [car]/). Oral feedback was provided during the practice session, but not during the experiment.

After the practice session, the gating paradigm started. All participants began with the consonant identification task, followed by the word identification task. There were short rest periods to prevent fatigue. The order of item presentation within each gated task (i.e., consonants and words) varied among the participants. Participants gave their responses orally and the experimenter wrote these down. The presentation of gates continued until the target item was correctly recognized on five consecutive presentations to avoid random guessing. If the target item was not correctly recognized, presentation continued until the end of the stimulus. After gated identification training, the post-test HINT scores were obtained. The first session finished at this point. Similar to Lidestam et al. (2014), after each training condition (audiovisual or auditory gated speech identification training, respectively), the participants rated the effort required for speech identification tasks on a questionnaire on a visual analog scale from 0 (no effort) to 100 (maximum effort).

One month after the first session, participants returned to the laboratory for the measurement of their HINT follow-up scores and obtaining their pure-tone hearing thresholds using an audiometer (Interacoustics AC40).

## Results

### Comparison of Gated Audiovisual and Auditory Speech Identification Tasks

**Table 2** shows the mean number of gates required for correct identification of consonants and words in the auditory and audiovisual modalities, respectively. The participants in the audiovisual training were able to identify both consonants and words with fewer gates (faster identification) than the participants in the auditory training group. These findings are in agreement with our prior studies that showed that the association of visual cues to auditory speech signal resulted in faster identification of consonants and words in aided hearing-impaired listeners (Moradi et al., 2016; Moradi et al., unpublished).

**Table 2:** Mean number of gates (with Standard Deviations in parentheses) required for correct identification of consonants and words in auditory and audiovisual modalities, respectively.

Type of gated task	Audiovisual	Auditory	Inferential statistics
Consonants	9.17 (1.81)	10.69 (1.56)	*t*(23) = 2.20, *p* = 0.038, *d* = 0.88
Words	12.65 (2.02)	14.22 (1.35)	*t*(23) = 2.21, *p* = 0.037, *d* = 0.89

### Effects of Gated Audiovisual and Auditory Speech Training on HINT Performance

**FIGURE 2:**
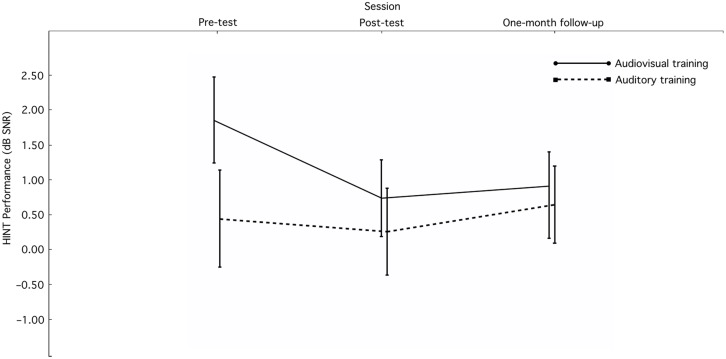
**HINT scores (*M* ± *SE*) for the experimental and control groups in the pre-test (before the training), post-test (promptly after the training), and one-month follow-up (1 month after the training)**.

**Figure 2** shows the mean scores and standard errors for HINT performance from before the training, promptly after the training, and from the one-month follow-up. Prior to training, the mean HINT scores in audiovisual and auditory training groups were 1.85 dB SNR (*SD* = 2.54) and 0.44 dB SNR (*SD* = 1.96), respectively, which means that the audiovisual training group had about 1.5 dB higher SNR in HINT than the auditory training group. However, there was no significant difference between the audiovisual and auditory training groups in HINT performance before the training, *t*(23) = 1.52, *p* = 0.14 (ns. *d* = 0.61).

A 2 (Training condition: audiovisual, auditory) × 3 (Session: pre, post-, follow-up) split-plot factorial analysis of variance (ANOVA) with repeated measures on the second factor was conducted to examine the effect of training conditions on HINT performance across sessions. Results showed the main effect of training condition was not significant, *F*(1,23) = 0.90, *p* = 0.35. In addition, the main effect of session was not significant, *F*(2,46) = 2.29, *p* = 0.11. The interaction between training condition and session was not also significant, *F*(2,46) = 1.99, *p* = 0.15.

In the next step of analysis, we analyze the data in a within-subject manner to test our *a priori* hypothesis that gated audiovisual speech training subsequently improved HINT performance and this improvement was retained after one-month follow-up; whereas there should be little or no improvement for the gated auditory training group.

### Pre- to Post-test Improvements and Pre- to One-month Follow-Up Test Maintenance

A one-way (Sessions [before, after, and one-month follow-up]) repeated measures ANOVA was conducted to examine the differences between HINT scores before and after the training in the experimental and control groups, respectively.

In the audiovisual training group, the main effect of sessions was significant, *F*(2,26) = 4.97, *p* = 0.015, $\eta_{p}^{2}$ = 0.28. Planned comparisons showed that audiovisual training improved sentence comprehension in noise ability promptly after the training (post-test vs. pre-test comparison), *t*(13) = 3.32, *p* = 0.006, *d* = 0.89. When comparing the HINT scores between one-month follow-up and before the training, the effect of audiovisual training in terms of improving sentence comprehension in noise was maintained, *t*(13) = 2.35, *p* = 0.035, *d* = 0.63. In addition, the difference between the post-test and one-month follow-up was not significant, *t*(13) = 0.43, *p* = 0.67 (ns. *d* = 0.12).

In the auditory training group, the main effect of session was not significant, *F*(2,20) = 0.31, *p* = 0.74 (ns. $\eta_{p}^{2}$ = 0.03). Planned comparisons also showed no effect of auditory training in terms of improving sentence comprehension in noise ability neither promptly after the training, *t*(10) = 0.35, *p* = 0.73 (ns. *d* = 0.11), nor at one-month follow-up, *t*(10) = 0.37, *p* = 0.72 (ns. *d* = 0.11).

### Self-effort Rating for the Identification of Gated Speech Training Tasks

The mean self-rated effort for the identification of gated speech tasks was 53.18 (*SD* = 13.09) in the auditory training group and 62.14 (*SD* = 20.64) in the audiovisual training group. A *t*-test comparison showed no significant difference between two groups in their subjective effort required for the identification of gated speech training tasks, *t*(23) = 1.25, *p* = 0.22. This finding corroborates Lidestam et al. (2014) by suggesting that the differences in HINT scores (particularly post-test scores) are not associated with subjective effort of participants in training conditions for the identification of speech stimuli.

## Discussion

The findings of the present study extend our previous studies in elderly hearing-aid users, by showing that prior exposure to gated audiovisual speech identification tasks subsequently improves auditory sentence identification in noise ability. In addition, this audiovisual speech training effect was independent of the idiosyncrasy of talkers as the talkers in the training materials and HINT, respectively, were different. Furthermore, for the first time, we showed that the effect of audiovisual speech training was retained 1 month after the training (the HINT performance was better at both post-test and one-month follow-up in comparison to the pre-test; and there was no difference between post-test and one-month follow-up). This suggests that gated audiovisual speech training can be used in a reliable aural rehabilitation program for people with hearing loss. Further, our findings are in line with those of Richie and Kewley-Port (2008), who showed that audiovisual vowel training improved auditory vowel recognition and the auditory identification of key words in sentences in participants with normal hearing. Together, our findings suggest that a sensory-rich speech training program comprising audiovisual speech training (containing complementary audio and visual speech cues) improves auditory identification of speech stimuli in people with hearing loss.

In the auditory training group, and despite providing an interfering noise during the training, there was no significant improvement in the HINT performance at neither post-test nor one-month follow-up when comparing to the pre-test. Most likely, this was due to using only one session of training in the present study. One may speculate that by providing several sessions of auditory training when background noise is added to training materials, then auditory training becomes an active training method (i.e., more challenging due to more degradation of the speech signal, see Rao et al., 2017) that subsequently would result in better HINT performance. In their review, however, Henshaw and Ferguson (2013) evaluated the effects of auditory training on aural rehabilitation of people with hearing loss. In terms of on-task improvement (i.e., improvement in a given speech task that was the exactly same task as the one trained), their review revealed that most of the studies reported significant on-task improvement after auditory training. In terms of far-task improvement (i.e., improvement in a speech task or a test that had not been directly trained), their review showed that auditory training studies have generally failed to show such a “far-transfer” improvement. Our lack of effect on auditory training is in agreement with the conclusion by Henshaw and Ferguson (2013). We used consonants and words in the training materials whereas the outcome measure was identification of sentences (in noise) in the HINT task, where no improvement was observed.

One explanation for the efficiency of this short audiovisual speech training program is that the gated audiovisual identification of consonants and words represents an active audiovisual speech identification training program. This is because it forces listeners to allocate their attention to the auditory and visual components of phonetic and lexical incoming speech signals and quickly map them onto their corresponding phonological and lexical structures in the brain. In addition, a background noise was added to the gated audiovisual speech identification training in both the present study and in that by Lidestam et al. (2014) in order to increase listeners’ attention (or cognitive effort) for pairing the auditory and visual components of incoming speech signals to create a coherent audiovisual speech token. According to the Ease of Language Understanding model (Rönnberg et al., 2013) working memory plays a critical role in perceiving speech stimuli in degraded listening conditions such as in background noise or in people with hearing loss (for an opposing view, see Füllgrabe and Rosen, 2016). Hence, an increase in cognitive effort (e.g., the addition of background noise) might to some extent reinforce the effect of gated audiovisual speech identification training (see Wayne and Johnsrude, 2012). In addition, we speculate that longer audiovisual training sessions would improve auditory sentence identification in noise ability even more. An alternative approach for providing longer sessions of audiovisual speech training could be provided in the form of “e-health rehabilitation,” by developing computer software, mobile applications (“apps”), or internet interventions, to be used at home by people with hearing loss.

We also suggest that audiovisual speech training in noise with multiple talkers could be another efficient training approach for improving speech-in-noise in people with hearing loss. In such a training condition, participants focus more on the visual component of the audiovisual speech materials for extracting phonetic features, in order to correctly identify those speech tokens. Simultaneously, they have to ignore the distractive speech from multiple talkers, which makes this task more cognitively demanding than simple audiovisual speech training in noise. A comparison between gated audiovisual speech training in background noise (as used in the present study) and audiovisual speech training in noise with multiple talkers would be a very interesting future research topic.

According to Shams and Seitz (2008), our interaction with the external world is usually multisensory rather than unisensory, and the human brain has evolved to process, operate, and learn optimally in multisensory rather than unisensory conditions. Consequently, the efficiency of training protocols would be optimized if they consisted of multisensory materials that are more approximate to natural settings. In fact, the ecological validity of audiovisual speech training is more evident for people with hearing loss as, due to their hearing loss, they rely more on visual speech cues to disambiguate the identity of a target speech signal than their counterparts with normal hearing when both auditory and visual speech cues are available (Walden et al., 1990; Desai et al., 2008).

In a magnetoencephalographic study, Zion Golumbic et al. (2013) revealed that audiovisual relative to auditory presentation enhanced the capacity of the auditory cortex to track the temporal speech envelope of the talker, particularly in “cocktail party” conditions. Similarly, Crosse et al. (2015) showed that cortical representation of the speech envelope was enhanced by congruent audiovisual presentation, even in a noise-free condition. In addition, Ganesh et al. (2014) in an electroencephalography study showed that early audiovisual presentation subsequently reduced the amplitude and latency of P2 response (a speech specific component that presumably is related to processing of physical characteristics of a speech sound prior to its categorization, see Näätänen and Winkler, 1999). The Ganesh et al. (2014) study in fact denotes that audiovisual relative to auditory presentation speeds up the auditory processing of speech stimuli for their identification. Further, Li et al. (2011), in a functional magnetic resonance imaging study, also showed that audiovisual relative to auditory-only presentation facilitated access to the neural representation of semantic content, in terms of both within-class reproducibility (discriminability of semantic content within the same semantic category) and between-class discriminability (discriminability of semantic content between two different semantic categories). In their review, Shams et al. (2011) suggested that multisensory training can boost subsequent unisensory processing, most likely because early exposure to multisensory stimuli quickly recalibrates unisensory maps in the brain, creates a new connection between unisensory cortical areas, or because the unisensory representation of stimuli (i.e., auditory-only or visual-only representation of stimuli) are integrated in a multisensory manner.

Together, we hypothesize that there are two mechanisms that, independently or together, account for the efficiency of gated audiovisual speech training on subsequent auditory identification performance. First, gated audiovisual speech training reinforces auditory routes for phonological and lexical representations in long-term memory (Shams et al., 2011), which subsequently facilitates access to those representations in an auditory-only modality condition (Li et al., 2011). In such a case, competing words in the target’s cohort are readily eliminated in subsequent sentence comprehension in noise by the previous audiovisual speech processing (training). Second, audiovisual speech training enhances the capacity of the auditory cortex to detect the perceptual changes in the target speech due to background noise (Zion Golumbic et al., 2013; Ganesh et al., 2014; Crosse et al., 2015); helping listeners to identify the target words at higher noise levels (i.e., lower SNRs).

The participants in the auditory training group may have been somewhat discouraged by their poorer performance as they needed longer exposure (i.e., higher number of gated) in auditory speech identification training tasks than the participants in the audiovisual training group. This might have had a negative influence on subsequent speech-in-noise task performance. Hence, the better performance in subsequent auditory speech-in-noise task in audiovisual training group was perhaps not solely due to the association of visual cues with auditory speech training materials. The effect may, to some extent, have been due to higher motivation and compliance caused by audiovisual speech training materials. Nevertheless, the results showed no significant difference in self-effort ratings for the training speech materials between audiovisual and auditory training groups. In addition, if the abovementioned argument was true, we believe that it is a merit for the use of audiovisual speech training over auditory speech training to improve participants’ motivation and compliance. Sweetow and Sabes (2010) showed that compliance with home-based auditory-only training programs in hearing-aid users was low, and most participants did not complete the training. The interaction between compliance and participation, the modality of speech training (auditory vs. audiovisually), and the amount of benefit provided by the training materials needs to be investigated in future studies.

Note that the data from the present study did not reveal that that the gated audiovisual speech training is better than auditory speech training since there were no significant differences in HINT scores between audiovisual and auditory training groups in each of three sessions (prior to the training, promptly post-training, and one-month follow-up). Future studies are needed to better evaluate audiovisual versus auditory speech training on subsequent on-task and far-task speech improvement and the compliance with the training programs in people with hearing loss.

### Idiosyncrasy of the Talkers

The idiosyncrasy of talkers is a key factor in perceptual learning studies, as previously acquired knowledge about a talker (obtained in the audiovisual training condition) should be available in the subsequent unisensory modality task (Schall et al., 2013; Schall and von Kriegstein, 2014; Schelinski et al., 2014). Riedel et al. (2015) suggested an “audiovisual feed-forward model” to explain how multisensory training with familiar persons subsequently improves the auditory-only speech recognition of those persons. According to this model, the human brain quickly and efficiently learns about “a new person” by his or her own auditory and visual characteristics that are relevant in the auditory-only or the visual-only identification conditions (forming an audiovisual simulation of that person). When a visual signal is lacking, this simulation feeds back to auditory areas and improves the auditory-only recognition (voice) of that person. In the present study and in our earlier studies, the talkers in the gated audiovisual training and auditory sentence comprehension in noise tests were not the same; hence, the audiovisual feed-forward model (Riedel et al., 2015) is at variance with our findings. We have coined the term “perceptual doping” to refer to the fact that the visual component of the audiovisual speech signal is much more distinct than the auditory-alone component for retuning (or setting up) phonological and lexical maps in the mental lexicon. As a consequence, the maps to phonological and lexical representations become more distinct and easier to access – *without effort*. These distinct and more accessible maps (doped, updated, or enriched maps) are maintained after the (gated) audiovisual speech training, which subsequently facilitates auditory route mapping to those phonological and lexical representations in the mental lexicon. The gating format presumably adds to the efficiency by its task characteristics. Successive repetition and extension of the audiovisual speech stimuli allow listeners to allocate their attention to more fine-grained phonological features, which are much easier to find in audiovisual format.

The perceptual doping hypothesis is also supported by neuroimaging studies which show that audiovisual relative to auditory-alone presentation improves the auditory route map for speech comprehension (Li et al., 2011; Zion Golumbic et al., 2013; Ganesh et al., 2014; Crosse et al., 2015). The exact encoding of visual characteristics of a given talker (audiovisual feed-forward model; Riedel et al., 2015) does not seem necessary for subsequent improvement in auditory-only speech recognition. The perceptual doping effect is more about the default mode of speech processing which is multisensory (Rosenblum, 2005; Ghazanfar, 2010) and its instant and maintained consequences on unisensory mapping of speech signal onto a linguistic representation in the brain and is generalizable across hearing status of individuals.

One limitation of the present study is the sample size in the audiovisual and auditory training groups. Since hearing-impaired listeners are more heterogeneous in perceiving speech stimuli than normal hearing listeners, we recommend that future studies evaluating training programs in people with hearing loss consider a larger sample size to detect significant differences that we could not achieve in the present study (e.g., interaction between group and session).

In the present study, we randomized the participants to the auditory or auditory training groups. Due to the heterogeneity of people with hearing loss in perceiving speech stimuli, we suggest that future studies allocate their participants to training groups based on matching instead of randomization. This is because prior to the training conditions in the present study, the audiovisual and auditory training groups differed around 1.5 dB SNR, which makes the interpretation the data more difficult.

## Conclusion

The findings of the present study highlight the efficiency of gated audiovisual speech training for improving auditory sentence identification in noise ability in elderly hearing aid users; furthermore, this efficiency persisted 1 month after the training. The present study did not show that the audiovisual speech training is better than auditory speech training (in terms of the between-groups comparison), however. A controlled comparison of audiovisual and auditory speech training on subsequent auditory improvement of speech stimuli using larger sample size is needed for future studies. In addition, we suggest examining the efficiency of in-home audiovisual speech training programs for the aural rehabilitation of hearing aid users, as they can offer longer periods of training that can boost auditory speech identification in people with hearing loss.

## Author Contributions

The present study was designed by the SM, BL, and AW collected the data. MH helped us in recruiting the participants based on inclusion and exclusion criteria. The data was analyzed with SM and BL with comments from the JR. The manuscript was written with SM, BL, and JR helped us in theoretical aspects of the manuscript. In addition, MH commented and helped in Materials and Methods section.

## Conflict of Interest Statement

The authors declare that the research was conducted in the absence of any commercial or financial relationships that could be construed as a potential conflict of interest.
